# Shortened Modified Look-Locker Inversion recovery (ShMOLLI) for clinical myocardial T1-mapping at 1.5 and 3 T within a 9 heartbeat breathhold

**DOI:** 10.1186/1532-429X-12-69

**Published:** 2010-11-19

**Authors:** Stefan K Piechnik, Vanessa M Ferreira, Erica Dall'Armellina, Lowri E Cochlin, Andreas Greiser, Stefan Neubauer, Matthew D Robson

**Affiliations:** 1 University of Oxford Centre for Clinical Magnetic Resonance Research (OCMR), Department of Cardiovascular Medicine, John Radcliffe Hospital, Oxford, UK; 2 Stephenson CMR Centre, Libin Cardiovascular Institute of Alberta, University of Calgary, Calgary, Canada; 3 Dept. of Physiology, Anatomy and Genetics, University of Oxford, Oxford, UK; 4 Siemens AG Healthcare Sector, Erlangen, Germany

## Abstract

**Background:**

T1 mapping allows direct *in-vivo *quantitation of microscopic changes in the myocardium, providing new diagnostic insights into cardiac disease. Existing methods require long breath holds that are demanding for many cardiac patients. In this work we propose and validate a novel, clinically applicable, pulse sequence for myocardial T1-mapping that is compatible with typical limits for end-expiration breath-holding in patients.

**Materials and methods:**

The Shortened MOdified Look-Locker Inversion recovery (ShMOLLI) method uses sequential inversion recovery measurements within a single short breath-hold. Full recovery of the longitudinal magnetisation between sequential inversion pulses is not achieved, but conditional interpretation of samples for reconstruction of T1-maps is used to yield accurate measurements, and this algorithm is implemented directly on the scanner. We performed computer simulations for 100 ms<T1 < 2.7 s and heart rates 40-100 bpm followed by phantom validation at 1.5T and 3T. *In-vivo *myocardial T1-mapping using this method and the previous gold-standard (MOLLI) was performed in 10 healthy volunteers at 1.5T and 3T, 4 volunteers with contrast injection at 1.5T, and 4 patients with recent myocardial infarction (MI) at 3T.

**Results:**

We found good agreement between the average ShMOLLI and MOLLI estimates for T1 < 1200 ms. In contrast to the original method, ShMOLLI showed no dependence on heart rates for long T1 values, with estimates characterized by a constant 4% underestimation for T1 = 800-2700 ms. *In-vivo*, ShMOLLI measurements required 9.0 ± 1.1 s (MOLLI = 17.6 ± 2.9 s). Average healthy myocardial T1 s by ShMOLLI at 1.5T were 966 ± 48 ms (mean ± SD) and 1166 ± 60 ms at 3T. In MI patients, the T1 in unaffected myocardium (1216 ± 42 ms) was similar to controls at 3T. Ischemically injured myocardium showed increased T1 = 1432 ± 33 ms (p < 0.001). The difference between MI and remote myocardium was estimated 15% larger by ShMOLLI than MOLLI (p < 0.04) which suffers from heart rate dependencies for long T1. The *in-vivo *variability within ShMOLLI T1-maps was only 14% (1.5T) or 18% (3T) higher than the MOLLI maps, but the MOLLI acquisitions were twice longer than ShMOLLI acquisitions.

**Conclusion:**

ShMOLLI is an efficient method that generates immediate, high-resolution myocardial T1-maps in a short breath-hold with high precision. This technique provides a valuable clinically applicable tool for myocardial tissue characterisation.

## Introduction

In cardiovascular magnetic resonance (CMR), tissue contrast is generated by a combination of intrinsic tissue properties such as spin-lattice (T1) and spin-spin (T2) relaxation times, and extrinsic properties such as imaging sequence and settings. Signal intensity in conventional CMR images is displayed on an arbitrary scale, and thus is not suited to comparison between subjects. T1-mapping provides a quantitative surrogate marker for the cellular environment of the myocardial water allowing direct comparison between patients and examinations, which can operate without the need for exogenous contrast agents.

T1 relaxation times depend on the composition of tissues, and each tissue type exhibits a characteristic range of normal values at a selected magnetic field strength [1]. Deviation from established ranges can thus be used to quantify the effects of pathological processes. Focal and global T1 changes are reported in a number of myocardial diseases, such as myocardial infarction [2,3], heart failure [4], valvular heart disease [5], and systemic diseases with cardiac involvement such as amyloidosis [6,7] or systemic lupus erythematosus [8]. T1-mapping may be a sensitive technique for detecting diffuse fibrosis in heart failure and valvular heart disease, which have been described by abnormal post-contrast T1 values but not by conventional late gadolinium enhancement (LGE) imaging [4,5].

**Figure 1:**
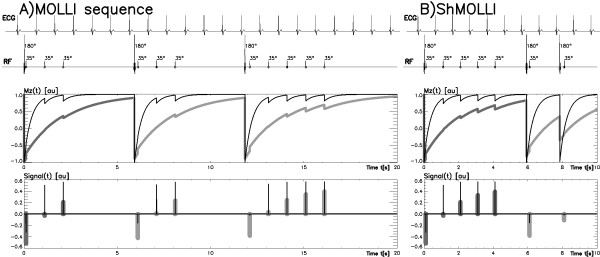
**ECG-gated pulse sequence schemes for simulation of A) MOLLI and B) ShMOLLI at a heart rate of 60 bpm**. SSFP readouts are simplified to a single 35° pulse each, presented at a constant delay time TD from each preceding R wave. The 180° inversion pulses are shifted depending on the IR number to achieve the desired first TI of 100, 180 and 260 ms in the consecutive inversion recovery (IR) experiments. The plots below represent the evolution of longitudinal magnetisation (Mz) for short T1 (400 ms, thin lines) and long T1 (2000 ms, thick lines). Note that long epochs free of signal acquisitions minimise the impact of incomplete Mz recoveries in MOLLI so that all acquired samples can be pooled together for T1 reconstruction. In ShMOLLI the validity of additional signal samples from the 2^nd ^and 3^rd ^IR epochs is determined by progressive nonlinear estimation.

An established method for myocardial T1-mapping is the modified Look Locker inversion recovery (MOLLI) pulse sequence [9]. It merges images from three consecutive inversion-recovery (IR) experiments into one data set (Figure 1A), generating single-slice T1 maps of the myocardium [9,10]. Clinical use of MOLLI was demonstrated in myocardial infarction [2,3]. One barrier to the clinical adoption of this method is the 17 heart beat breath-hold required to obtain a single slice T1 map. This is usually acceptable for normal subjects who, on average, can hold their breath for 20.9 s (range 13-74 s) in end-expiration [11] (the respiratory phase in which CMR is usually performed). However, patients with pulmonary compromise could only achieve such breath holds for 9.1 s (range 2-16 s) [11]. Long breath holds are problematic in the elderly population due to frequent respiratory comorbidities [12] and in patients on beta-blockers where lower heart rates translate into breath holds of over 20 seconds. Therefore, a significant improvement in speed over the MOLLI method is required to achieve T1 mapping for sequential slice by slice whole heart coverage in routine clinical practice.

We hypothesise that it is possible to determine T1 maps of the heart with high precision in a short breath-hold. We thus present a shortened alternative to MOLLI (ShMOLLI) which can generate rapid and high-resolution myocardial T1-maps in a single short breath-hold of only 9 heartbeats. We investigate the accuracy of T1 measurements by ShMOLLI against MOLLI in simulation and gel phantom experiments over a wide range of heart rates and T1 values. The findings are validated *in-vivo *for normal human myocardium at 1.5T and 3T. To extend the *in-vivo *validation range we also present findings in four human subjects with recent myocardial infarction and four normal subjects after gadolinium contrast.

## Materials and methods

### Simulation and sequence design

Simulations were performed in IDL (Interactive Data Language ver. 6.1, ITT Visual Information Solutions) by implementing equations for the piece-wise calculation of longitudinal magnetisation (Mz(t)) and the signal samples generated by a train of arbitrarily-spaced ideal excitation pulses. Inversion pulses are assumed to be perfect 180° excitations; readouts are simulated as single pulses of 35°. Both sequences had three IR epochs. Simulations were performed for MOLLI based on its optimised variant [13], which collects 3+3+5 samples in three consecutive IR epochs separated by long recovery periods, which are approximated as being fully recovered in the reconstruction. The shortest effective TI [9] in each IR are 100, 180 and 260 ms; incremented by heart beat period for subsequent samples (Figure 1A). ShMOLLI uses a similar effective TI principle but collects only 5+1+1 samples and IR epochs are separated by only one T_RR _(R-R interval), which does not approximate to full recovery of the magnetization (Figure 1B).

Simulations using simplified pulse sequences outlined in Figure 1 were performed for T1 ranging from 50 to 2700 ms (50 ms increments) and for HR between 40-100 bpm (20 bpm increments). For each combination of parameters, we analysed 200 sample trains with noise representative of our phantom measurements.

### Processing

**Figure 2:**
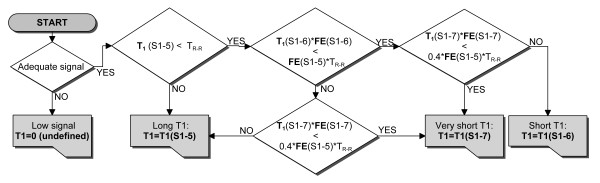
**ShMOLLI conditional data analysis**. Simplified algorithm for inclusion of samples to circumvent the impact of short recovery epochs in T1 estimation. "FE" is the fit error calculated as the square root of the sum of squared residuals divided by number of samples minus one. "S1-5" denotes the set of samples from the first inversion recovery, "S1-6" and" S1-7" are supplemented by samples from consecutive IR experiments. T_R-R _is a heart beat interval.

Significant shortening of the recovery epochs means that Mz can be severely affected by preceding IR epochs in the excitation sequence as demonstrated for long T1 values in Figure 2B (thick grey line). Consequently, the signal samples from the 2^nd ^and 3^rd ^IR of ShMOLLI do not fit the simple method of pooling data together into a single IR equation as outlined for MOLLI [9] and would produce significant errors if used in reconstruction. This problem is circumvented by conditional data analysis according to the algorithm presented in Figure 2. In essence, in regions of long T1, samples 1-5 are fitted using [9]. For shorter T1 (0.4T_RR _< T1 < T_RR_) samples 1-6 are used. For very short T1 (< 0.4T_RR_), samples 1-7 are utilised. As the T1 is not known accurately the inclusion thresholds 0.4T_RR _and T_RR _are modified depending on the fit error.

### On-the-scanner implementation

The MOLLI and ShMOLLI acquisitions used a research sequence provided by Siemens (Siemens Healthcare, Germany) utilising the optimised parameter set for MOLLI [13] and ShMOLLI. T1 s were fitted using the algorithms described above with an AMOEBA optimisation method [14]. This reconstruction fitting was coded in C++ directly into the scanner's multi-threaded parallel processing image pipeline. As a result, T1-maps are available for viewing in about 10 seconds following image acquisition.

### Phantom studies

Fifteen 50 ml Agarose and NiCl gel phantoms [15] with T2~50 ms and T1 ~100-2500 ms were studied at 1.5T (Avanto, Siemens Healthcare, Germany) using a 32 channel body array and at 3T (Trio, Siemens Healthcare) using a 16 channel body array. The artificially generated ECG scanner triggers were used to validate HR range from 40-100 bpm with 10 bpm increments. Imaging parameters were the same for MOLLI and ShMOLLI: FOV = 300 × 300 mm, voxel size = 1.17 × 1.17 × 8 mm, matrix = 256 × 256 (interpolated from 128 × 128 acquisition matrix, 95 phase encoding steps), flip angle = 35°, TR/TE = 1.96/0.98 ms.

Reference T1 relaxation times were calculated offline based on images collected using slice-selective IR with non-segmented spin echo readout. TI = 33, 100, 300, 900, 2700, 5000 ms. TR/TE = 10 s/6.3 ms; 80 phase encoding steps with total image acquisition time of 13 minutes. Sizing and positioning were identical to the studied MOLLI methods. Regions of interest were placed in each tube using an automated method and reference T1 s were fitted per pixel and a mean T1 determined.

### *In-vivo* studies

Ethics approval was granted for all study procedures and informed consent was obtained from all subjects.

#### Human volunteers

10 normal volunteers (7 men; age 35 ± 7 years, normal ECGs without history of cardiac diseases or symptoms) underwent CMR imaging at 1.5T and 3T on the same day. Following standard planning, end-expiration basal, mid-cavity and apical short-axis images using MOLLI and ShMOLLI were collected. Images for specific TI were collected using exactly the same SSFP readouts for both methods to allow direct comparisons, typically: TR/TE = 2.14/1.07 ms, flip angle = 35°, FOV = 340 × 255 mm, matrix = 192 × 144, 107 phase encoding steps, interpolated voxel size = 0.9 × 0.9 × 8 mm, GRAPPA = 2 with 24 reference lines, cardiac delay time TD = 500 ms; 206 ms acquisition time for single image. A single slice, which was judged to have the "best quality" at the time of scanning, was repeated twice at the end of the protocol to assess short-term intra-scan variability of the T1 measurements; this was not performed in the first pilot case. Offline post-processing involved manual tracing of endo- and epi-cardial contours for analysis of the T1 measurements in myocardial segments 1 to 16 of the American Heart Association (AHA) 17-segment model [16] using in-house software.

#### Gadolinium contrast application

Matching pairs of ShMOLLI and MOLLI pre-contrast and post-contrast T1 maps were obtained in 4 female subjects (61 ± 3 years old) without pre-existing cardiac disease who underwent a separate research protocol at 1.5T. Subjects underwent adenosine stress perfusion at 140 μg/kg/min for 3 min, followed by a bolus of Gd (Gadodiamide, Omniscan, GE Healthcare, Amersham, UK, 0.03 mmol/kg body weight). After 20 minutes, resting perfusion imaging was performed using 0.03 mmol/kg of Gd followed immediately by a top-up Gd of 0.10 mmol/kg for LGE imaging. Matching T1-maps were obtained at baseline and ~14 minutes after adenosine stress perfusion. Finally, 4 pairs of images were collected before, and one after, the LGE images. The dynamic evolution of T1 recovery after the final Gd bolus was corrected with 3^rd ^order polynomial for the purpose of constructing Bland-Altman plots.

#### Patients with recent myocardial infarction

4 patients (3 men; age 53 ± 10 years) underwent CMR at 3T following the diagnosis of a first acute ST-elevation type myocardial infarction (STEMI) post primary percutaneous coronary intervention (PCI). LGE images were obtained 24-48 hours post acute infarct [17]. T1-maps using ShMOLLI and MOLLI were obtained 5-17 days after the ischemic event at a single representative slice replicating settings from the normal control volunteer study. Manual contouring of the endo- and epicardium was followed by calculation of the distribution of T1 values within the defined myocardium. The resulting distributions were clearly bi-modal and were fitted using a two-component Gaussian model in order to assess T1 in injured and unaffected myocardium.

### Statistics

Unless stated otherwise, the results are presented as mean ± SD/mixed SD, where the "mixed SD" is estimated as the independent combination of average individual SD and the interindividual SD. For estimation of the relative variability we use the coefficient of variation (CV = 100%*SD/Mean). The term "noise penalty" is used to describe the expected relative increase in CV resulting from predicable factors, i.e. the reduced number of samples. Significance of the differences between population means is calculated using 2-tailed Student T-tests, paired whenever possible, and quoted when p <0.05.

## Results

### Simulation and phantom studies

**Figure 3:**
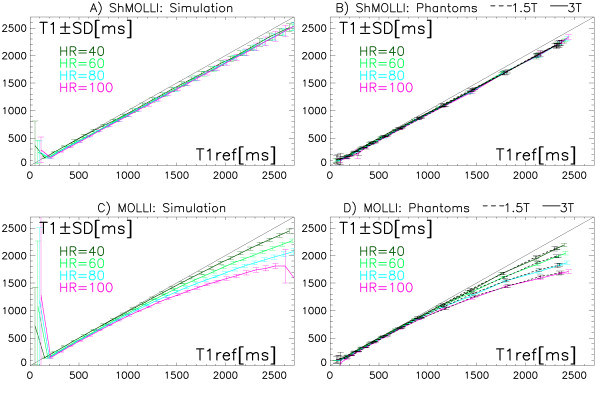
**Relationship between T1 measurements for ShMOLLI (top row) and MOLLI (bottom row) and the corresponding reference T1 (T1ref) depending on selected simulated heart rates (HR)**. Diagonal line represents the ideal identity line. Simulation (A&C) and Phantom (B&D) measurements in 3T and 1.5T (dashed lines) overlap and are in close agreement with simulation results. NOTE. The lines are offset by 15 ms horizontally for each HR value to reduce the overlap between lines.

The accuracy of both methods is dependent on the T1 and heart rate. Generally, as HR and T1 increase, the T1 measurements diverge increasingly from the ideal identity line (Figure 3). This is observed in both simulation (Figure 3A, C) and phantom measurements with excellent agreement at both field strengths (Figure 3B, D). Figure 3C, D demonstrate a clear dependence of MOLLI estimates on HR, with relative underestimation reaching -30% for the highest HR and longest T1 studied. In contrast, ShMOLLI calculation (Figure 3A, B) demonstrates tight overlap of the values with significant reduction in dependence on the heart rate. For T1 s longer than approximately 800 ms the deviation of ShMOLLI estimates from the identity line is proportional to T1. The corresponding relative error of underestimation by ShMOLLI in this range is effectively a constant of -4%.

The variability of T1 measurements across all simulations or pixels is shown as whiskers in Figure 3. For long T1 s, measurements by ShMOLLI are based on 5 samples whereas MOLLI measurements are based on 11 samples. Therefore ShMOLLI has a predicted noise penalty of 48% versus MOLLI (√(11/5)). For shorter T1 s, 6 or 7 TI samples are used with a predicted noise penalty of 35% and 25%. Inclusive of the heavily nonlinear processing, for the reference T1 range of 300-2600 ms, we find the simulated average CV is 2.7% for ShMOLLI and 2.1% for MOLLI, translating to an overall 28% noise penalty for using ShMOLLI. In phantom studies, the noise penalty for ShMOLLI is 21% at 3T (Figure 3B) and 61% at 1.5T (Figure 3C).

### Normal human subjects

**Figure 4:**
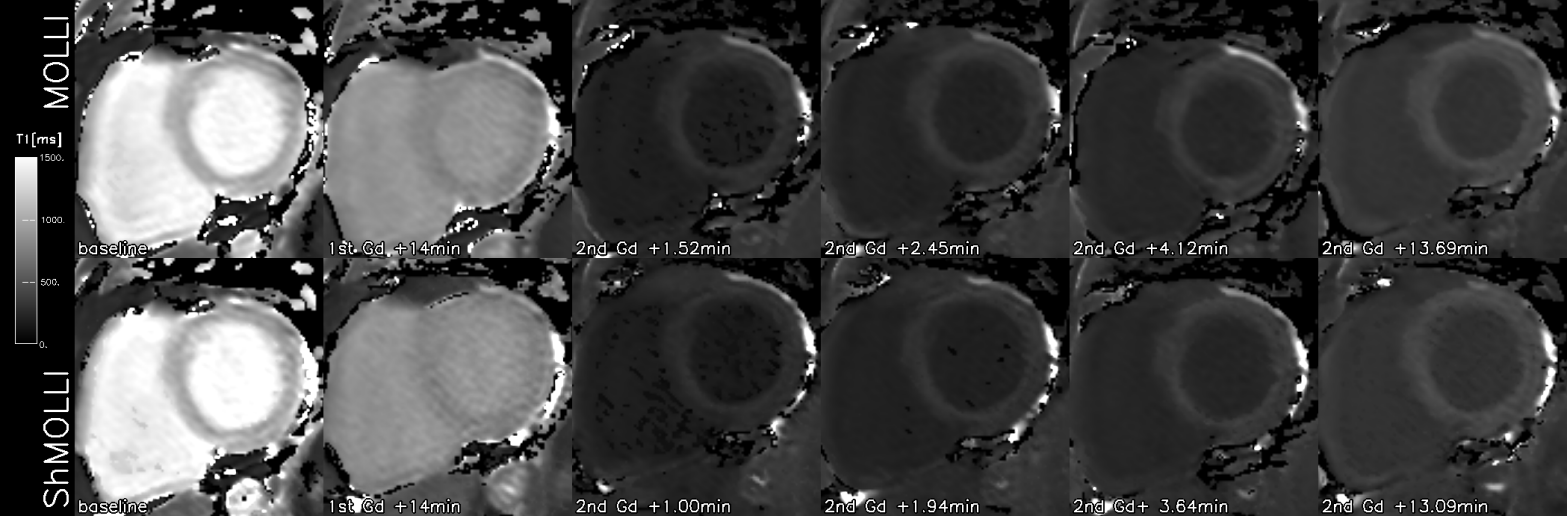
**Representative short axis slice T1 maps of the normal myocardium obtained using MOLLI (top row) and ShMOLLI (bottom row) at 1.5T at the baseline and following Gd administration for perfusion imaging (1st Gd) and after top-up (2nd Gd) at times shown in panel labels**.

In normal control volunteers, the myocardial ShMOLLI and MOLLI T1-maps did not show any visual differences (Figure 4). The imaging time was 9.0 ± 1.1 s and 17.6 ± 2.9 s, respectively. Both methods utilise the same imaging SSFP sequence and suffered from occasional localised image artefacts. These were more severe at 3T where the maximum CV over the worst single segment reached 79% for MOLLI and 47% for ShMOLLI. Aside from the extreme outliers, the accuracy of T1 measurements benefited both from the increase in field strength and the number of samples included. CV was below 10% in 97% of the segments for MOLLI and 94% for ShMOLLI at 3T. At 1.5T the proportions were 89% and 85%, respectively. The median CVs were 4.7% (MOLLI, 3T), 5.5% (ShMOLLI, 3T), 6.1% (MOLLI, 1.5T) and 7.4% (ShMOLLI, 1.5T). Overall, ShMOLLI carried a 10% noise penalty at 1.5T (p < 0.001) but was the same as MOLLI at 3T (3%, p = 0.8). The lack of statistical significance in the latter comparison was attributed to outliers, which was addressed by removing 3 (out of 160) data points from further analysis. This returned the expected differences (p < 0.001) between methods indicating an 18% noise penalty for ShMOLLI at 3T and 14% at 1.5T. There was a clear benefit of using high field MR in a 33% reduction in CV for MOLLI and 24% for ShMOLLI (p < 0.001, both differences) despite the use of a better coil array at 1.5T.

**Figure 5:**
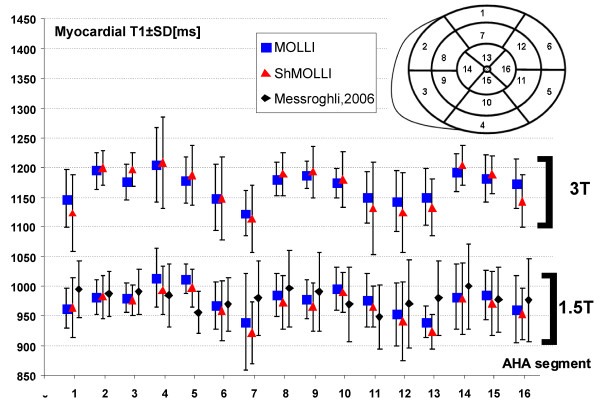
**T1 values for AHA myocardial segments 1-16 (see insert) at 1.5T and 3T using ShMOLLI and MOLLI**. Results of current study as compared to previously published values at 1.5T [10].

The average T1 values for myocardial segments derived using both ShMOLLI and MOLLI at 1.5T and 3T are presented in Figure 5. At 1.5T, average myocardial T1 s by ShMOLLI were 966 ± 48/88 ms and 976 ± 46/80 ms by MOLLI. T1 values for normal myocardium by both methods compared closely to previously published T1 values at 1.5T [10]. At 3T, average myocardial T1 s were similar for ShMOLLI and MOLLI (1166 ± 60/91 ms and 1169 ± 45/73 ms).

**Figure 6:**
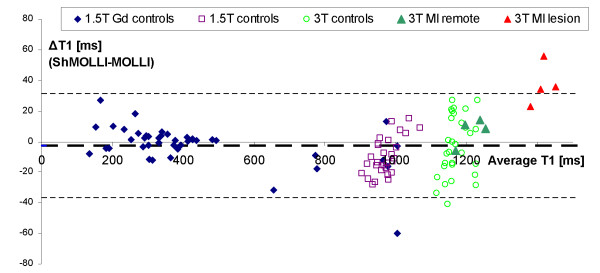
**Bland-Altman plot shows good agreement between the average ShMOLLI and MOLLI myocardial T1 values pooled across our material obtained at 1.5T and 3T**. Note that the distribution of differences at approximately ± 17 ms is also representative of the repetition accuracy for either method. Note that the majority of outliers relate to the infarct data at 3T where the demonstrated MOLLI bias dominates over noise considerations even at normal heart rates. Thick dashed line represents overall average difference between measurements (-3 ms), thin dashed lines represent 2SD range.

Analysis of differences between methods (Figure 6) revealed that myocardial ShMOLLI T1 s were shorter than MOLLI T1 s by 10 ± 16 ms (relative 1 ± 1.6%, p < 0.001) at 1.5T. At 3T both methods produced similar values (Δ =-0.2 ± 16 ms or 0.02 ± 1.3%, p = 0.13). Given that the average HR was 63 ± 8 bpm (1.5T) and 61 ± 7 bpm (3T), for the closest emulated HR of 60 bpm, we predict from phantoms the difference between methods to be -12.5 ms (for normal myocardial T1 = 1000 ms at 1.5T) and +0.5 ms (for normal myocardial T1 = 1200 ms at 3T). Analysis of intra-scan variability showed that the average repetition error was similar to the spread of differences between methods, approximately ± 16 ms (~1.6%) between any pair of repeated measurements at 1.5T. At 3T the intra-scan variability was ± 29 ms (2.4%) for ShMOLLI and ± 76 ms (7%) for MOLLI. The latter was due to the 3 myocardial segments characterised by excessive CV of over 38%. When these were removed, MOLLI repetition accuracy improved to ± 18 ms (1.5%).

### Gadolinium contrast application

None of the subjects demonstrated LGE lesions. The baseline T1 values were 982 ± 28 ms, comparable with other controls at 1.5T. Depending on the dose of gadolinium and time point the T1 showed expected shortening with the lowest average myocardial T1 of 135 ± 33 ms and subsequent recovery towards normal values (Figure 4). The differences between the ShMOLLI and MOLLI measurements are shown together with the remaining *in-vivo *data in Figure 6 and confirm good agreement *in-vivo *in the low T1 regime predicted in simulation and phantom measurements.

### Patients with recent myocardial infarction

**Figure 7:**
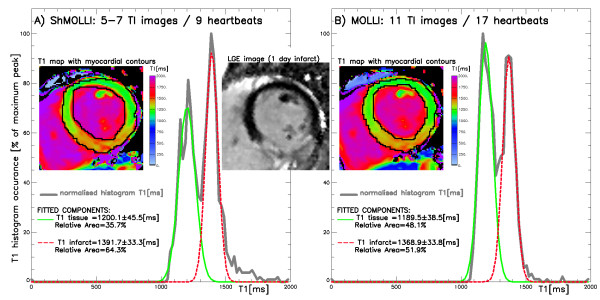
**The distribution of T1 in case #1 at 3T using A) MOLLI and B) ShMOLLI reveals two distinct populations of values within the myocardium, which can be fitted using two Gaussian curves, corresponding to injured (long T1) and unaffected (normal T1) myocardium**. Regions of increased T1 demonstrate spatial co-localisation with LGE 4 days earlier (image insets).

As in normal controls, the image acquisition in patients was much faster using ShMOLLI (9.9 ± 1.6 s) than with MOLLI (18.3 ± 3.0 s) without visually perceivable impact on the reconstructed myocardial T1 maps. Figure 7 shows an example of recent transmural inferior infarct 4 days following primary PCI, as demonstrated by LGE at 3T. Table 1 shows T1 values for injured and unaffected normal myocardium measured by distinct T1 distribution peaks in all 4 cases. Unaffected myocardium T1 values showed no significant difference from those of healthy volunteers at 3T measured by ShMOLLI (1216 ± 42 ms, p = 0.09) and MOLLI (1209 ± 35 ms, p = 0.1). Injured myocardium had increased T1 (p < 0.001) of 1432 ± 33 ms (ShMOLLI) and 1396 ± 27 ms (MOLLI). The relative difference in T1 values between injured and unaffected myocardium were 17.8 ± 6% (ShMOLLI) and 15.5 ± 5% (MOLLI), which was statistically different between methods (p < 0.04). The width of identified T1 distribution peaks, indicative of noise penalty, was on average 20% larger for ShMOLLI than for MOLLI (p < 0.02). The area of injured myocardium as identified by T1-maps was on average 4 ± 10% (range -3 to +19%) larger than the area identified by LGE.

**Table 1:** Assessment of myocardium by T1-mapping and LGE at 3T in acute STEMI treated by 1° PCI.

Case	Gender (age years)	T1-mapping	T1 [ms]		Injured myocardium[%]	
		Method	*Unaffected*	*Injured*	*T1-based*	*LGE (days prior)*
1	F(50)	ShMOLLI	1200 ± 46	1392 ± 33	64%	39%(-4d)
		MOLLI	1189 ± 39	1369 ± 34	52%	

2	M(49)	ShMOLLI	1269 ± 69	1425 ± 21	50%	44%(-14d)
		ShMOLLI	1244 ± 50	1418 ± 30	38%	
		MOLLI	1248 ± 47	1391 ± 21	47%	

3	M(68)	ShMOLLI	1244 ± 51	1445 ± 45	58%	48%(-16d)
		MOLLI	1230 ± 33	1389 ± 46	44%	

4	M(45)	MOLLI	1171 ± 47	1433 ± 48	41%	46%(-14d)
		ShMOLLI	1165 ± 55	1469 ± 59	40%	

	**53 ± 10**	***Averages***	**1217 ± 37**	**1424 ± 23**	**48 ± 9%**	**44 ± 4%**

## Discussion

**Figure 8:**
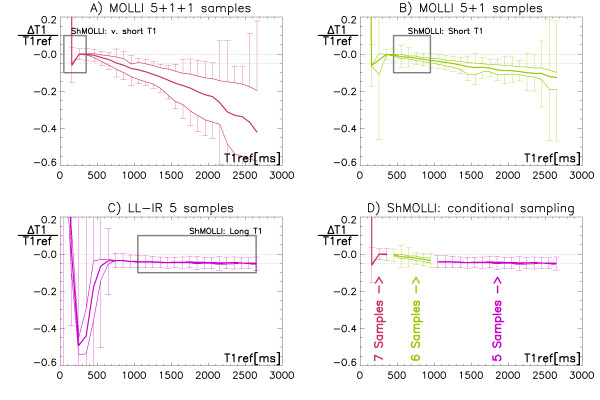
**Simulated average relative T1 estimation error (thick lines) with boundaries characterising dependence on heart rate (thin lines) and measurement noise (whiskers) as a function of reference T1ref**. A) Standard MOLLI reconstruction applied to the proposed ShMOLLI sampling scheme demonstrates very large errors reaching -60%, with accuracy acceptable only within the shortest T1 range (grey rectangle). B) Accuracy is improved for short T1 range when the last sample is removed from analysis; however very short T1estimates suffer increased variability and the longer T1 are affected by heart rate dependent bias and noise. C) Simple Look-Locker IR experiment has adequate accuracy only for long T1. D) Concept of conditional use of the marked parts of reconstructions A/B/C to obtain the wide range of ShMOLLI T1 estimates with the average bias within 5% range (dotted lines).

To exploit benefits of quantitative T1 mapping we sought to develop an accurate practical method characterised by a shorter breath-hold of the order of 10 seconds, which is easily achievable for patients. Figure 8A demonstrates that such drastic shortening of the MOLLI sampling scheme results in significant errors when a straightforward T1 reconstruction [9] is used. The novelty in ShMOLLI approach arises from simulations prior to this study, where we observed that just a single Look-Locker IR is sufficient to accurately estimate long T1 values that are longer than the T_RR _[18] (Figure 8C). Conversely, inclusion of additional samples from subsequent IRs is essential for the accurate estimation of short T1 values but does not require long epochs for complete recovery of longitudinal magnetisation. Thus, our final design of ShMOLLI is based not only on an abbreviated TI sample set and a drastic reduction of the recovery periods between subsequent inversion recovery experiments. Side effects of such rapid sampling are compensated with a novel concept of conditional data processing to distinguish between long and short T1 relaxation times in order to optimally utilise the available TI samples for accurate non-linear T1 estimation over a wide range of T1 (Figure 8D).

We used both methods to measure a wide range of T1 applicable to 1.5 and 3T, encompassing values corresponding to contrast enhanced myocardium (T1~150-500 ms), fat (T1~200 ms), liver (~700 ms), skeletal muscle and myocardium (~1000 ms), blood (~2000 ms) and lymph or pericardial effusion (~3000 ms). ShMOLLI demonstrates robust T1 measurement properties across a wide range of T1 values and heart rates in simulation and phantoms. While both ShMOLLI and MOLLI slightly underestimate true T1 values, MOLLI shows prominent dependency on heart rate. An empirical correction for MOLLI has been proposed that addresses specifically the normal myocardial T1 values at 1.5T [3,10]. In contrast, ShMOLLI underestimation is consistently ~4% for any T1 above 800 ms. This permits easier adjustment to calculate the true T1 values in wide range independently of heart rate:

$$T_{1}^{True}=\frac{T_{1}^{Measured}}{1+relativeError}\approx 1.04\cdot T_{1}^{Measured}$$

Excluding any such corrections (as no corrected values are presented in this work), the measurements of myocardial T1 by ShMOLLI at 1.5T are in good agreement with in-vivo data published in the literature [19], particularly with previous measurements using the MOLLI sequence [10]. The observed minor differences between the two methods conform closely to simulation and phantom validation.

The purpose of the limited *in-vivo *material was to directly compare ShMOLLI performance with MOLLI over a wide range of clinically relevant scenarios. This included measurements in normal myocardium, following shortening of T1's as a result of contrast administration and T1 prolongation related to myocardial infarction. T1 measurements agreed in healthy controls within ± 17 ms noise boundaries for normal myocardium and after Gd administration. Our MI data is consistent with previously published reports [2,9,13,20] showing that the area of infarct demonstrates increased T1 values on non-contrast T1 maps. LGE images were acquired 4-16 days prior to T1-maps, which precludes direct comparisons of infarct size assessment by LGE imaging and T1-mapping. Nonetheless, it is clear that the distribution of T1 values within the myocardium shows two distinct peaks of normal and long T1 s, the latter co-localising to the area of injured myocardium as seen on LGE. ShMOLLI showed a 15% larger T1 difference between injured and unaffected myocardium, which can be directly attributed to its superior quantitation of long T1 s.

### Noise considerations

Typically, the penalty for reducing the number of images collected for T1 reconstruction is increased variability within the resulting T1 maps. Our simulation and phantom measurements predicted the noise penalty for ShMOLLI to be as much as 61%. However, in-vivo T1 measurements by ShMOLLI showed noise penalty of only 14% (1.5T) and 18% (3T) as compared to MOLLI T1 s. The repetition error was 1.6% for both methods at 1.5T and only excluding the worst measurements brought out the expected noise advantage of MOLLI at 3T. We found that while MOLLI benefited from more samples for the majority of measurements, the higher SNR was bought at the cost of more prominent outliers, especially at 3T. This may be due to the ShMOLLI conditional processing acting as an error checker, but detailed analysis of such effects exceeds the scope of this work. Overall, for most of the T1 values tested, the in-vivo noise penalty for ShMOLLI due to a shorter imaging time is only 10-20%. We attribute this favourable observation mostly to the beneficial effect of the short breath-hold, which limits the incidence of breathing motion, estimated as the cause for 31% of image artefacts [10]. The small reduction in precision of ShMOLLI T1 maps in-vivo is an excellent trade-off for halving the imaging time and breath-hold, rendering the ShMOLLI both accurate and clinically acceptable.

The ShMOLLI technique holds promise for wider application of non-contrast T1-mapping towards cardiac conditions in which there are focal changes, such as myocardial infarction and myocarditis. It may be pivotal in studying conditions in which there are diffuse myocardial changes, such as chronic heart failure, hypertrophic cardiomyopathy and infiltrative diseases such as cardiac amyloidosis and sarcoidosis. The presented comparisons warrant the ongoing ShMOLLI deployment on its own merit in several more comprehensive patient validation studies.

## Conclusion

The novel ShMOLLI sequence for myocardial T1-mapping generates robust, high resolution quantitative T1 maps in agreement with published data in the literature in just 9 heart-beats across a wide range of heart rates and T1 values. Single short breath holds are typical for routine examinations to make them easily achievable for patients and permit wider clinical application of quantitative mapping. Implementation of nonlinear T1 fitting directly in the scanner image reconstruction pipeline yields immediate access to T1-maps for viewing, allowing for re-acquisition if necessary. In patients presenting with recent myocardial infarction, preliminary data demonstrates ShMOLLI superiority in distinguishing injured from normal myocardium, with areas of long T1 co-localizing with injured myocardium as assessed by LGE. ShMOLLI provides a valuable clinically applicable tool for myocardial tissue characterisation with or without the use of contrast agents.

## Competing interests

US patent pending **61/387,591**: SKP, MDR and AG. *SYSTEMS AND METHODS FOR SHORTENED LOOK LOCKER INVERSION RECOVERY (Sh-MOLLI) CARDIAC GATED MAPPING OF T1*. September 29, 2010.

## Authors' contributions

SKP provided the concept and implementation of the method, performed simulations, phantom measurements, data analysis and drafted the manuscript. VMF collected and processed data in normal controls and contributed very significantly to the drafting of the manuscript. EDA collected patient datasets. LEC helped with design of phantoms. AG contributed to the sequence implementation on the scanner. SN contributed to study design acting as the last author from clinical viewpoint. MDR contributed to the study concept, design, method implementation and the manuscript as last author. All authors read, commented or edited the manuscript, and approved the final version.
